# Propofol for endotracheal intubation in neonates: a dose-finding trial

**DOI:** 10.1136/archdischild-2019-318474

**Published:** 2020-01-13

**Authors:** Ellen H.M. de Kort, Sandra A. Prins, Irwin K.M. Reiss, Sten P. Willemsen, Peter Andriessen, Mirjam M. van Weissenbruch, Sinno H.P. Simons

**Affiliations:** 1 Neonatology, Maxima Medical Center, Veldhoven, The Netherlands; 2 Neonatology, Erasmus MC-Sophia Children's Hospital, Rotterdam, The Netherlands; 3 Neonatology, Amsterdam UMC location VU Medical Center, Amsterdam, The Netherlands; 4 Biostatistics, Erasmus Medical Centre, Rotterdam, The Netherlands

**Keywords:** pharmacology, analgesia, neonatology, pain

## Abstract

**Objective:**

To find propofol doses providing effective sedation without side effects in neonates of different gestational ages (GA) and postnatal ages (PNA).

**Design and setting:**

Prospective multicentere dose-finding study in 3 neonatal intensive care units.

**Patients:**

Neonates with a PNA <28 days requiring non-emergency endotracheal intubation.

**Interventions:**

Neonates were stratified into 8 groups based on GA and PNA. The first 5 neonates in every group received a dose of 1.0 mg/kg propofol. Based on sedative effect and side effects, the dose was increased or decreased in the next 5 patients until the optimal dose was found.

**Main outcome measures:**

The primary outcome was the optimal single propofol starting dose that provides effective sedation without side effects in each age group.

**Results:**

After inclusion of 91 patients, the study was prematurely terminated because the primary outcome was only reached in 13% of patients. Dose-finding was completed in 2 groups, but no optimal propofol dose was found. Effective sedation without side effects was achieved more often after a starting dose of 2.0 mg/kg (28%) than after 1.0 mg/kg (3%) and 1.5 mg/kg (9%). Propofol-induced hypotension occurred in 59% of patients. Logistic regression analyses showed that GA and PNA did not predict effective sedation or the occurrence of hypotension.

**Conclusions:**

Effective sedation without side effects is difficult to achieve with propofol and the optimal dose in different age groups of neonates could not be determined. The sedative effect of propofol and the occurrence of hypotension are unpredictable and show large inter-individual variability in the neonatal population.

**What is already known on this topic?**

- Endotracheal intubation is stressful and painful and causes multiple adverse effects.
- Non-emergency endotracheal intubation in neonates should always be performed with the use of premedication.
- Despite its off-label use, the lack of clear dosing guidelines and concerns about safety, propofol is widely used in this context.

**What this study adds?**

- Effects and side effects of propofol are highly variable and unpredictable with extensive interindividual variability.
- Relatively high doses are needed to provide effective sedation and propofol carries a high risk of hypotension, even when initial low doses are used.
- Propofol in the neonatal population should be used with careful consideration.

## Introduction

As awake intubation has multiple harmful effects,^1–7^ the routine use of premedication before non-emergency intubation in neonates has become standard of care.^8–11^ However, there is insufficient knowledge and lack of consensus about the most effective and safe strategy. Propofol is considered one of the acceptable options, despite being off-label for use in newborns, gaps in knowledge regarding optimal dosing and concerns about safety.^12^ Because of its rapid onset and recovery, and its ease of use, propofol as a sedative for endotracheal intubation has been implemented into clinical practice in several neonatal intensive care units (NICUs).^13–17^ Previous trials studying propofol have shown conflicting results on sedative effect and concerning effects on blood pressure.^16–19^

Used propofol starting doses range from 1.0 to 2.5 mg/kg, with cumulative doses ranging from 1.0 to 6.0 mg/kg for successful intubation.^15–19^ Although gestational age (GA) and postnatal age (PNA) are important determinants of propofol pharmacokinetics,^20^ fixed propofol starting doses are often used for the entire neonatal population regardless of GA and PNA. The Exploratory Propofol Dose-Finding Study In Neonates (NEOPROP) is the only available dose-finding study in newborns that recently determined the effective propofol dose in 50% (EC^_50_^) of patients for three different GA groups. This study also showed a great decrease in mean arterial blood pressure and 62% incidence of hypotension.^21^

It is crucial to find propofol doses that are safe and effective in the entire newborn population. Therefore, we performed the NEOPROP-2 trial, which aimed to find age-specific propofol starting doses that provide effective sedation without side effects in neonates.

## Methods

### Study design and setting

A prospective multicentre dose-finding study was conducted at three level III NICUs in the Netherlands between July 2014 and January 2018. An interim analysis was planned after every 6 months of inclusion, by an independent data and safety monitoring committee. The parents of all included patients provided written informed consent.

### Participants

**Figure 1:**
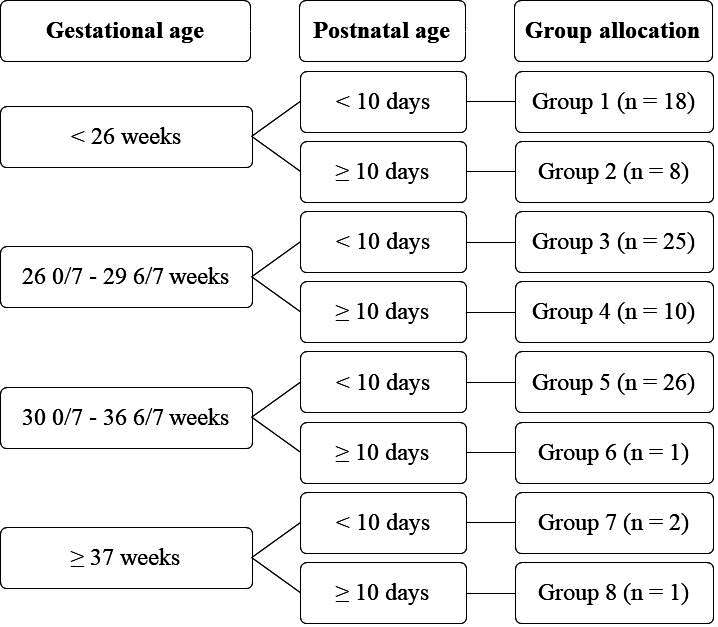
Group allocation.

Neonates were eligible if they had a PNA of <28 days and required non-emergency endotracheal intubation. Exclusion criteria were major congenital anomalies or neurological disorders, upper airway anomalies, sedative or opioid administration in the preceding 24 hours and previous inclusion in the trial. The use of propofol was left to the discretion of the attending physician. If the haemodynamic status was judged to be sufficiently stable to use propofol, the patient could be included. Patients were stratified into eight different groups by GA and PNA (figure 1), based on expected variation in effect and propofol clearance.

### Interventions

#### Intubation procedure

Propofol was used as standard of care for endotracheal intubation in our units. Propofol (Fresenius Kabi, Schelle, Belgium) was administered as intravenous bolus followed by a saline flush for a total duration of 30 s. After propofol administration, the pre-intubation sedation level was assessed every 30 s up to 3 min after the infusion, using the Intubation Readiness Score (IRS).^22^ When the pre-intubation sedation level was adequate, intubation was continued. In the case of insufficient pre-intubation sedation level after 3 min, additional propofol was administered until the pre-intubation sedation level was sufficient. The amount of each additional propofol dose was left at the discretion of the attending physician. After intubation, the quality of intubation was measured by the Viby-Mogensen intubation score.^23^ Data regarding propofol doses and intubation attempts were reported.

#### Dose-finding approach

A sample size of five patients per dose per group was used, based on the large interindividual variability in effects of propofol that was found previously.^17^ The first five patients in every group received a starting dose of 1.0 mg/kg propofol. Based on these five patients, the dose was increased or decreased with 0.5 mg/kg in the next five patients up to a maximum starting dose of 3.5 mg/kg because of expected toxicity. If needed for further optimisation, a change of 0.25 mg/kg was applicable in the final dose-finding stage. Once the optimal propofol dose had been found, it was confirmed in another five patients. Dose finding was completed per group when the optimal propofol dose was found, or when the maximum starting dose of 3.5 mg/kg was reached.

### Primary outcome measures

The primary outcome of the study was the optimal single propofol starting dose for intubation in neonates with different GAs and PNAs defined as the single starting dose that provided effective sedation without significant side effects. Effective sedation was determined with two variables that both needed to be adequate: pre-intubation sedation level and quality of intubation. Pre-intubation sedation level was determined with the IRS, and adequate pre-intubation sedation level was defined as IRS 3 or 4.^22^ Quality of intubation was measured by the Viby-Mogensen intubation score.^23^ Good quality of intubation was defined as a score of ≤2 on each of the five items.

Predefined side effects included hypotension, myoclonus, chest wall rigidity, persistent respiratory and/or circulatory failure and bronchospasm. Blood pressure was measured invasively if an indwelling arterial catheter was present. Data were collected every minute from 5 min before until 30 min after the start of propofol administration, every 5 min from 30 to 60 min and every hour thereafter up to 24 hours. When no arterial catheter was present, blood pressure was measured non-invasively by an appropriately sized cuff every 5 min from 5 min before until 60 min after propofol administration and every hour thereafter until 24 hours. Propofol-induced hypotension was defined as a mean blood pressure (MBP) below postmenstrual age (PMA) detected in the first hour after propofol administration. Treatment of hypotension was left to the discretion of the treating physician.

For the primary outcome both effective sedation and absence of serious side effects needed to be positive. When either sedation was not effective or tthere were serious side effects, the primary outcome was not reached. Since both items needed to be positive, in case of one negative item and one missing item, the primary outcome was also not reached.

### Secondary outcome measures

Secondary outcomes were the optimal propofol starting dose in the entire study population (regardless of age group), the need for additional doses of propofol and side effects in the entire study population and sedative effect and side effects in the most frequently used propofol starting doses. Finally, a logistic regression analysis was performed to find potential variables predicting the sedative effect and side effects after propofol.

### Statistical analysis

The predefined sample size depended on which propofol dose was found to be adequate in five consecutive patients per group. Data were analysed using SPSS (IBM SPSS Statistics for Windows, V.22.0. Armonk, New York, USA), and R V.3.5 (R Core Team, Vienna, Austria). Patients were analysed according to the intention-to-treat principle. Baseline characteristics were described by percentages for qualitative variables and median (IQR) for quantitative variables. Comparison between dosing groups was performed with the Mann-Whitney U test for continuous variables and the Pearson’s χ^2^ test or the Fisher’s exact test, as appropriate, for categorical variables. Logistic regression analysis was performed to identify factors influencing the sedative effect and side effects of propofol with primary outcome, effective sedation and hypotension as outcome variables. We analysed the effects of gestational age (weeks), birth weight <10th percentile, male gender, postnatal age (hours) and propofol starting dose (mg/kg) on primary outcome and effective sedation. Total amount of propofol (mg/kg) was added as a confounder in the logistic regression analysis with hypotension as outcome variable. We used the Firth’s method to reduce the bias in logistic regression that arises as a consequence of the relatively small sample size.^24 25^

## Results

### Study population

The study population consisted of 91 patients (see table 1). Three patients were included despite their PNA exceeding 28 days (two patients in group 2 (39 and 32 days) and one patient in group 4 (29 days)).

**Table 1:** Patient characteristics

Characteristic	Entire study population (n=91)	Group 1 (n=18)	Group 2 (n=8)	Group 3 (n=25)	Group 4 (n=10)	Group 5 (n=26)	Group 6 (n=1)	Group 7 (n=2)	Group 8 (n=1)
Gestational age (week), median (IQR)	27.7 (25.9–30.6)	25.3 (24.8–25.6)	24.8 (24.4–25.6)	28.0 (26.9–28.9)	26.8 (26.3–27.1)	32.0 (30.4–33.6)	30.7	38.1	37.9
Birth weight (g), median (IQR)	1045 (825–1560)	720 (634–864)	740 (716–791)	1045 (890–1223)	938 (886–1076)	1678 (1480–2129)	1290	3845	2865
Birth weight <10th percentile, n (%)	23 (25)	5 (28)	0	8 (32)	1 (10)	8 (31)	1 (100)	0	0
Postnatal age (hour), median (IQR)	29 (9–213)	129 (38–173)	405 (384–681)	10 (6–31)	371 (307–524)	20 (5–26)	432	50	593
Actual weight (g), median (IQR)	1100 (830–1560)	705 (643–838)	923 (825–1084)	1065 (910–1193)	1105 (925–1123)	1678 (1460–2125)	1400	3848	3100
Male gender, n (%)	58 (64)	12 (67)	7 (88)	13 (52)	6 (60)	16 (62)	1 (100)	2 (100)	1 (100)
Reason for intubation, n (%)									
RDS	44 (48.4)	2 (11.1)	0	17 (68)	0	23 (88.5)	0	2 (100)	0
Apnoea	19 (20.9)	9 (50)	5 (62.5)	2 (8)	3 (30)	0	0	0	0
Sepsis/NEC	11 (12.1)	5 (27.8)	1 (12.5)	3 (12)	1 (10)	1 (3.8)	0	0	0
Respiratory insufficiency	13 (14.3)	1 (5.6)	2 (25)	3 (12)	4 (40)	2 (7.7)	0	0	1 (100)
Elective*	2 (2.2)	0	0	0	1 (10)	0	1 (100)	0	0
Other	2 (2.2)	1 (5.6)	0	0	1 (10)	0	0	0	0
Propofol starting dose (mg/kg), n (%)									
0.5	1 (1)	1 6)	0	0	0	0	0	0	0
1.0	30 (33)	4 (22)	5 (63)	5 (20)	6 (60)	6 (23)	1 (100)	2 (100)	1 (100)
1.5	23 (25)	5 (28)	3 (37)	5 (20)	4 (40)	6 (23)	0	0	0
1.75	9 (10)	0	0	5 (20)	0	4 15)	0	0	0
2.0	26 (29)	6 (33)	0	10 (40)	0	10 (38)	0	0	0
2.5	2 (2)	2 (11)	0	0	0	0	0	0	0

*Including prior to surgery or tube exchange.

NEC, necrotising enterocolitis; RDS, Respiratory Distress Syndrome.

### Study termination

An interim analysis after inclusion of 91 patients demonstrated a low inclusion rate in several groups and a 59% incidence of hypotension. In two age groups a propofol dose that provided effective sedation was found but caused hypotension in the majority of patients. Therefore, an optimal dose as predefined in the primary outcome in these two groups was not established. The study was prematurely terminated, therefore, in consultation with the data safety monitoring committee.

### Primary outcome

**Figure 2:**
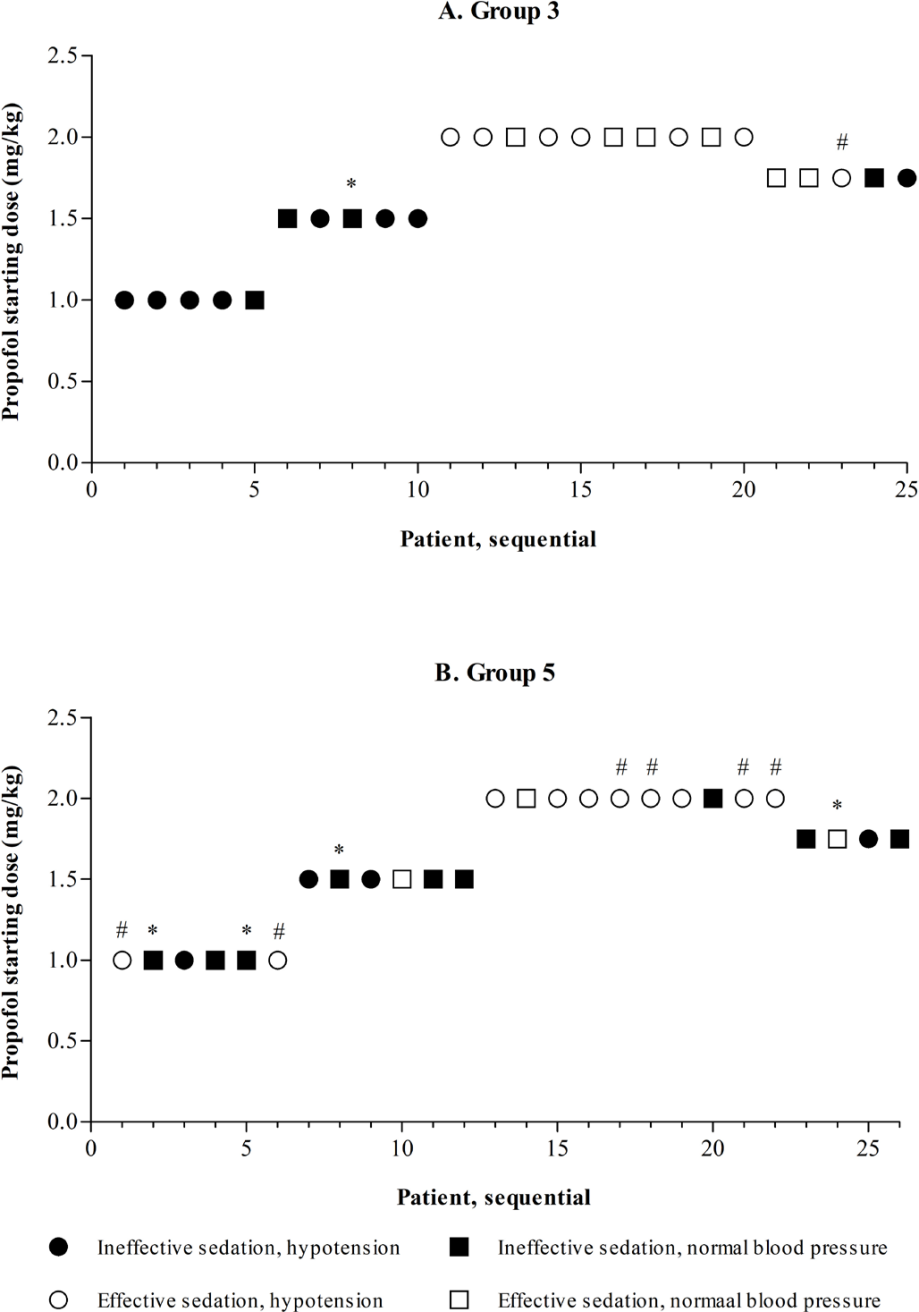
Dose finding in groups 3 and 5. *Missing data on blood pressure, in the dose-finding approach considered to be normal in the absence of evidence of hypotension; #missing data on intubation score, effective sedation only judged by pre-intubation sedation level. In group 5, the 1.0 and 1.5 mg/kg dosing subgroups both contain six instead of five patients. This was due to initial uncertainty of the suitability of the data for the primary outcome in one patient in both subgroups. An extra patient in both groups was included to ensure of total of five patients with viable data on the primary outcome. After re-evaluation the data of all six patients in both subgroups turned out to be suitable.

Dose finding was only completed in groups 3 and 5, without finding an optimal propofol dose. The results of the dose-finding approach in sequential patients in groups 3 and 5 are presented in figure 2. In both groups, starting doses of 1.0 and 1.5 mg/kg almost never led to effective sedation. A starting dose of 2.0 mg/kg led to effective sedation in many patients, but also led to a high incidence of hypotension, even after confirming this dose in another five patients per group. The dose, therefore, was decreased to 1.75 mg/kg, which did not provide effective sedation in the majority of patients in both groups.

### Secondary outcomes

In the entire study population, effective sedation without side effects was achieved in only 12 patients (13%). Additional propofol was administered in 65 patients (71%) and the median cumulative propofol dose for successful intubation was 3.0 mg/kg (range 1.0–6.0 mg/kg, IQR 2.0–3.75).

Propofol starting doses of 1.0, 1.5 and 2.0 mg/kg were used in further in-depth analyses. There were no differences in patient characteristics between the three groups, with the exception of PNA (table 2). This was lower in the 2.0 mg/kg dosing group, due to the higher inclusion numbers at younger postnatal ages. A starting dose of 2.0 mg/kg much more often led to effective sedation than starting doses of 1.0 and 1.5 mg/kg. The incidence of hypotension, however, was not different between the three starting doses.

**Table 2:** Patient characteristics and outcomes in three starting doses

	Dosing groups			Comparison between groups		
	1.0 mg/kg (n=30)	1.5 mg/kg (n=23)	2.0 mg/kg (n=26)	1.0 vs 1.5	1.0 vs 2.0	1.5 vs 2.0
*Patient characteristics*						
Gestational age (week), median (IQR)	27.5 (25.86–30.93)	26.86 (25.57–30.14)	29.07 (26.43–31.71)	P=0.37	P=0.66	P=0.20
Birth weight (g), median (IQR)	1075 (784–1410)	908 (780–1600)	1215 (895–1568)	P=0.46	P=0.51	P=0.19
Postnatal age (hour), median (IQR)	156 (12–397)	37.35 (21–387)	19.58 (8–43)	P=0.68	P=0.01	P=0.04
Male gender, n (%)	22 (73)	12 (52)	18 (69)	P=0.16	P=0.77	P=0.25
*Propofol dosing*						
Extra propofol administered, n (%)	25 (83)	20 (87)	11 (42)	P=1.0	P=0.002	P=0.002
Cumulative propofol dose (mg/kg), median (IQR)	3.0 (1.9–4.0)	3.4 (2.5–4.5)	2.0 (2.0–3.0)	P=0.06	P=0.97	P=0.03
*Primary outcome*						
No. of patients with data available	30 (100)*	23 (100)†	25 (96)‡			
Effective sedation without side effects, n (%)	1 (3)	2 (9)	7 (28)	P=0.57	P=0.02	P=0.15
*Sedative effect of propofol*						
Adequate pre-intubation sedation level, n (%)	7 (23)	7 (30)	24 (92)	P=0.75	P<0.001	P<0.001
Quality of intubation						
No. of patients with data available	3 (10)	7 (30)	19 (73)			
Good quality of intubation	1 (33)	3 (43)	18 (95)	P=0.18	P=0.02	P=0.003
Effective sedation						
No. of patients with data available	28 (93)	23 (100)	21 (81)			
Effective sedation, n (%)	1 (4)	3 (13)	18 (86)	P=0.21	P<0.001	P<0.001
*Hypotension*						
No. of patients with data available	24 (80)	21 (91)	26 (100)			
Occurrence of hypotension, n (%)	15 (63)	11 (52)	16 (62)	P=0.55	P=1.0	P=0.57
Volume resuscitation, n (% of hypotensive patients)	7 (47)	4 (36)	12 (75)	P=0.86	P=0.18	P=0.09

*Both patients with missing data on effective sedation had side effects and all six patients with missing data on side effects had insufficient sedation. Therefore, a conclusion on the primary outcome could be drawn for all 30 patients.

†Both patients with missing data on side effects had inadequate sedation and, therefore, a conclusion on the primary outcome could be drawn on all 23 patients.

‡In four patients with missing data on effective sedation, side effects were present and in only one patient both data on effective sedation and side effects were missing. Therefore, a conclusion on the primary outcome could be drawn in 25 out of 26 patients.

Sufficient MBP data after propofol administration were available for 82 patients (90%). Propofol-induced hypotension occurred in 48 (59%) patients. Of these, 26 patients (54%) were treated with volume resuscitation. Therapy with inotropes was started in nine patients (10%) at a median of 298 min after propofol administration (IQR 125–917 min). In seven of these patients, inotropes were started >2 hours after the start of propofol administration. In two other patients, inotropes were started within 2 hours and the hypotension is probably attributable to propofol. Comparison of MBP data before and after propofol was possible in 80 patients (88%). MBP decreased with a median of 34% (95% CI 36.5% to 29.1%) compared with baseline MBP. The lowest MBP was measured at a median of 21 min (95% CI 19.3 to 26.2 min).

Other side effects occurred in 10 patients (11%), including myoclonus in 8 patients (9%), bronchospasm in 1 patient (1%) and vocal cord spasm in 1 patient (1%). A total of 15 patients (16%) died at a median of 12 days after inclusion in this trial (range 0–57 days). Twelve patients died >72 hours after inclusion in the trial. One patient died from sepsis within 24 hours, and two patients died from necrotising enterocolitis between 24 and 48 hours after inclusion. None was judged as directly attributable to the propofol administration.

The results of the logistic regression analysis (table 3) showed that GA and PNA did not influence the effectiveness or safety of propofol.

**Table 3:** Logistic regression analysis with different outcome variables: primary outcome, effective sedation and hypotension

	Primary outcome (n=89)			Effective sedation (n=83)			Hypotension (n=82)		
	OR	95% CI	P value	OR	95% CI	P value	OR	95% CI	P value
Gestational age (weeks)	0.94	0.77 to 1.15	0.54	1.00	0.80 to 1.23	0.98	1.09	0.94 to 1.26	0.26
Birth weight <10th percentile (yes/no)	0.85	0.22 to 3.34	0.82	1.07	0.27 to 4.27	0.93	1.55	0.52 to 4.25	0.47
Male gender (yes/no)	1.72	0.46 to 6.40	0.42	2.67	0.76 to 9.36	0.13	0.94	0.36 to 2.45	0.90
Postnatal age (in hours)	1.00	0.99 to 1.00	0.69	1.00	0.99 to 1.00	0.65	1.00	0.99 to 1.00	0.45
Starting dose of propofol (mg/kg)	4.50	0.92 to 22.11	0.06	57.04	7.58 to 429.49	<0.001	1.09	0.35 to 3.39	0.88
Cumulative dose of propofol (mg/kg)							0.74	0.48 to 1.14	0.18

Corrected by Firth’s method to reduce bias because of relatively small sample size.

## Discussion

This dose-finding trial was designed to find the optimal single propofol dose for non-emergency endotracheal intubation providing effective sedation without significant side effects in neonates of different GAs and PNAs. To the best of our knowledge, this is the largest drug dose-finding study performed in the neonatal population. Unfortunately, dose finding could only be completed in two of the eight defined age groups without determination of the optimal propofol dose. Our results show a dose-dependent relationship for propofol to reach effective sedation. However, we also found the sedative effect to be unpredictable in the individual patient, and propofol is associated with a high incidence of hypotension. Based on these results, propofol might probably be not the most suitable premedication prior to endotracheal intubation in all neonates.

In contrast to our results, Smits *et al* were able to calculate specific propofol doses for preterm newborns in the first days of life that increased with GA.^21^ Their suggested propofol doses were lower than the doses that resulted in adequate sedation in our study. This difference could be explained by different ways of analyses and outcome parameters in both studies. We did not calculate the EC_50_, but showed that 2.0 mg/kg propofol starting dose is effective in 86% of patients.

The available literature shows conflicting results on the sedative effect of propofol. Doses of 1.0 and 2.5 mg/kg are found to provide sufficient sedation in some studies,^16 18^ while other studies found insufficient sedation with doses of 1.0, 2.0 and 2.5 mg/kg.^17 19^ These conflicting data underline that the sedative effect of propofol is difficult to predict. The indication for intubation could also play a role. For the Intubation–SURfactant–Extubation (INSURE) procedure, duration of sedation should be very short.^26^ Therefore, clinicians might accept lower levels of sedation to diminish the risk of insufficient respiratory drive after the administration of surfactant and, therefore, the inability to immediately extubate the patient. However, regardless of the procedures that follow intubation, the act of laryngoscopy is equally stressful and equal levels of sedation should in our opinion be pursued.

GA and PNA are known covariates in propofol pharmacokinetics.^20^ Therefore, we hypothesised that infants of different GAs and PNAs would need different propofol doses. Logistic regression analysis did not show a statistically significant effect of GA and PNA on the outcomes effective sedation and hypotension. Although unclear, the extended interindividual variability in the effect of propofol seems much more important than GA and PNA in predicting the effect. Titrating propofol until the desired effect is achieved in the individual patient is probably the only way to ensure effective sedation in every patient. This, however, might still lead to a high incidence of hypotension.

Propofol is known for its pronounced effect on blood pressure in the neonatal population. We found a median decrease in MBP of 34%, which is in accordance with other studies.^16 17 21^ The incidence of hypotension of 59% was comparable to that reported by Smits *et al* (64%),^21^ but much higher than founded by Welzing *et al* (38%).^16^ This could be explained by the much smaller study sample, the lower dosages and the different definition of hypotension.^16^ Ghanta *et al* did not report hypotension.^18^ This could be explained by the possibility that MBP measurements were not continued long enough to detect hypotension, as hypotension appears at a median of 10–20 min after propofol.^16 17^ Because of the pronounced effect that propofol can have on blood pressure, the haemodynamic status of the patient should be carefully evaluated before propofol is administered. In case of (impeding) haemodynamic compromise, other premedication with less pronounced effects on blood pressure should be considered.

Although blood pressure decrease after propofol is marked and there is a high incidence of hypotension, the implications for the short-term and long-term outcome are unclear. Blood pressure alone is a poor indicator of cardiovascular status.^27^ In 95% of patients in the dose-finding study by Smits *et al*, cerebral autoregulation was intact during episodes of hypotension.^28^ In the absence of clinical signs of shock, they labelled these episodes of hypotension as permissive.^21^ Two other small studies on the cerebral effects of propofol in the neonatal population also showed no important correlation between blood pressure and cerebral oxygenation.^29 30^ Although these findings are certainly reassuring, there is insufficient evidence on the short-term and long-term consequences of propofol-induced hypotension and blood pressure decrease to draw final conclusions. Until this is clarified in further studies, we should in our opinion be careful with designating propofol-induced hypotension and blood pressure decrease as permissive. On the other hand, the negative effects of propofol must be set against the negative effects of other premedication strategies. Almost all opioids, hypnotics and muscle relaxants also carry a risk of hypotension, and with fentanyl and remifentanil, there is also a risk of chest wall rigidity.^12^

Our study has several limitations. First, we were unable to perform dose finding as planned because patient inclusion in several groups proved to be very difficult. Reasons were insufficient time for achieving parental consent, and the very low incidence of endotracheal intubation in the higher gestational age groups. Second, we used a very strict definition of hypotension. Even a single measurement of MBP below PMA in the first 60 min after propofol was marked as hypotension. It is questionable whether this single measurement of MBP below PMA has any clinical relevance. Adding a time element to the definition may better reflect the patients with clinically relevant hypotension. Unfortunately, we were unable to provide synchronised neuromonitoring data, which could have helped to study the clinical relevance of hypotension on cerebral oxygenation and perfusion in greater detail. Third, the treatment of hypotension was left to the discretion of the treating physician, which is likely to have caused variability between clinicians and between centres.

## Conclusions

The results of this large dose-finding study suggest that in the neonatal population it is difficult to achieve effective sedation without the occurrence of significant side effects with a single propofol bolus. The effects and side effects of propofol in the neonatal population are highly variable and unpredictable. Propofol in the neonatal population should only be used after careful consideration in each individual patient and should be titrated based on the sedative effect with strict monitoring of blood pressure and haemodynamic status. As long as the ideal premedication strategy in the neonatal population has not been elucidated, the pros and cons of different strategies including propofol should be balanced against each other. A greater effort should be made to move forward from a one-strategy-fits-all idea towards personalised neonatal pharmacology.
